# Incidence, risk factors, and clinical characteristics of airway complications after lung transplantation

**DOI:** 10.1038/s41598-023-27864-1

**Published:** 2023-01-12

**Authors:** Hyeon Hwa Kim, Kyung-Wook Jo, Tae Sun Shim, Wonjun Ji, Jee Hwan Ahn, Dong Kyu Oh, Sang-Bum Hong, Jae Kwang Yun, Geun Dong Lee, Sehoon Choi, Dong Kwan Kim, Seung-Il Park, Ho Cheol Kim

**Affiliations:** 1 Division of Pulmonology and Critical Care Medicine, Department of Internal Medicine, Asan Medical Center, University of Ulsan College of Medicine, 88 Olympic-Ro 43-Gil, Songpa-Gu, Seoul, 05505 Republic of Korea; 2 Department of Thoracic and Cardiovascular Surgery, Asan Medical Center, University of Ulsan College of Medicine, Seoul, Republic of Korea

**Keywords:** Risk factors, Respiratory tract diseases

## Abstract

Airway complications may occur after lung transplantation and are associated with considerable morbidity and mortality. We investigated the incidence, risk factors, and clinical characteristics of these complications. We retrospectively reviewed the medical records of 137 patients who underwent lung transplantation between 2008 and 2021. The median follow-up period was 20 months. Of the 137 patients, 30 (21.9%) had postoperative airway complications, of which 2 had two different types of airway complications. The most common airway complication was bronchial stenosis, affecting 23 patients (16.8%). Multivariable Cox analysis revealed that a recipient’s body mass index ≥ 25 kg/m^2^ (hazard ratio [HR], 2.663; *p* = 0.013) was a significant independent risk factor for airway complications, as was postoperative treatment with extracorporeal membrane oxygenation (ECMO; HR, 3.340; *p* = 0.034). Of the 30 patients who had airway complications, 21 (70.0%) were treated with bronchoscopic intervention. Survival rates did not differ significantly between patients with and without airway complications. Thus, our study revealed that one fifth of patients who underwent lung transplantation experienced airway complications during the follow-up period. Obesity and receiving postoperative ECMO are risk factors for airway complications, and close monitoring is warranted in such cases.

## Introduction

Lung transplantation is the final therapeutic option for end-stage lung disease, and it improves survival and quality of life^1,2^. The number of lung transplantations has been increasing worldwide; the most common indications for lung transplantation are, in order, interstitial lung disease, chronic obstructive pulmonary disease (COPD), cystic fibrosis, and pulmonary hypertension^3^. Lung transplantation is being increasingly performed in Korea, and the outcomes there are comparable with other published data^4^. Although the outcomes of lung transplantation have improved over time, with some differences depending on specific lung diseases, the median length of survival is still only approximately 6–7 years^3,5^.

Airway complication is a major complication that must be overcome for improved outcomes after lung transplantation, and the reported incidence of airway complications has varied widely (approximately 2–30%)^6,7^. Various types of airway complications, including dehiscence, granulation, airway stenosis, tracheobronchomalacia, bronchial fistula, and anastomotic infection, have afflicted lung transplant recipients^6^, and are associated with considerable morbidity^8^. Although the exact mechanism of airway complication still needs to be elucidated, postoperative impaired blood flow in the donor bronchus, an inevitable consequence of disruption of the dual arterial supply to the donor’s lung during lung harvesting, has been regarded as a major cause of airway complication^9–11^. Because revascularization of the donor’s lung typically takes 2–4 weeks, the circulation of the donor bronchus is dependent on retrograde low-pressure collateral flow from the pulmonary artery during this period^9,12,13^. Therefore, the airway is vulnerable to ischemia during the immediate postoperative period, and most airway complications occur within the first year of transplantation^7,13^. However, over the years, several surgical techniques have been advanced to improve the postoperative bronchial ischemia, from telescoping anastomoses, end-to-end anastomoses, to shortening of the donor bronchus, and the incidence of airway complications has decreased^11,14–16^. In addition, several other risk factors associated with the development of anastomotic ischemia and airway complications have been previously investigated; donor–recipient height mismatch, primary graft dysfunction, acute rejection, colonization with *Aspergillus fumigatus*, use of sirolimus in the early postoperative period, and prolonged ventilator care^13,17–20^. However, optimal surgical techniques remain controversial, and risk factors reported in several previous studies are inconsistent^13,17,18^. Moreover, despite recent significant improvements in surgical techniques, donor/recipient selection, and perioperative management, airway complications continue to be a major cause of morbidity. We therefore investigated the incidence, risk factors, and clinical characteristics of airway complications after lung transplantation.

## Methods

### Study population and design

For this single-center, retrospective study, we reviewed the data of patients who underwent bilateral lung transplantation at Asan Medical Center, Seoul, Republic of Korea, between October 2008 and June 2021. We excluded patients who underwent multiorgan transplantation (12 who received heart–lung transplants and 2 who received lung-liver transplants). Patients were divided into two groups: those who experienced airway complications after transplantation and those who did not experience any airway complication during the follow-up period.

Data on demographics and clinical characteristics were extracted from the medical records. Recipient-related variables included age, sex, body mass index (BMI), height, diseases that necessitated transplantation, preexisting pulmonary hypertension (assessed by echocardiography or invasive right heart catheterization), smoking status, prior thoracic surgery (either lung biopsy or lobectomy), preoperative infectious episodes, preoperative bronchial colonization (*Pseudomonas aeruginosa, Aspergillus*) (defined as the repeated identification of the microbes in sputum or bronchoalveolar lavage culture with three or more positive samples during the year prior to transplantation), use of corticosteroids and immunosuppressants, duration of mechanical ventilation and extracorporeal membrane oxygenation (ECMO) therapy, and length of intensive care unit (ICU) stay. Donor-related variables included age, sex, and height.

Donor-to-recipient size mismatching was defined as cases in which the donor’s predicted total lung capacity (pTLC) was less than or equal to the recipient’s pTLC^21,22^. We calculated pTLC according to the prediction equations of the European Coal and Steel Community^23^. Preoperative steroid usage was assessed with a stratified daily dose of methylprednisolone or equivalent within a month before transplantation. The study protocol was approved by the Institutional Review Board of the Asan Medical Center, Ulsan University College of Medicine (IRB No. 2021-1324) in accordance with guidelines of the Declaration of Helsinki. Informed consent was waived by Institutional Review Board of Asan Medical Center due to retrospective nature of the study, and all methods were performed in accordance with the relevant guidelines and regulations.

### Perioperative management and follow-up protocol

Before surgery, all patients received induction immunosuppression therapy consisting of a high dose of intravenous methylprednisolone and basiliximab; maintenance treatment comprised standard triple immunosuppression therapy with tacrolimus, mycophenolate mofetil, and methylprednisolone^4^. Methylprednisolone 500 mg was administered intravenously at the time of transplantation, 375 mg the next day, and 2 mg/kg/day for the next two days, and then the daily dose was reduced to 1 mg/kg/day. The dose was gradually tapered to a goal of prednisolone 5 mg/day over several months to one year, during which intravenous methylprednisolone was converted to oral prednisolone at 0.5 mg/kg/day. A target trough level of tacrolimus was 10–15 ng/mL for the first 6 months and 8–12 ng/mL thereafter. In cases of renal impairment caused by tacrolimus, conversion from tacrolimus to sirolimus was considered. The target trough level of mycophenolate mofetil was 1–3 µg/mL.

Several drugs were administered to prevent infection. To prevent *Pneumocystis jiroveci* infection, trimethoprim/sulfamethoxazole, 80/400 mg, was administered daily. To prevent cytomegalovirus infection, ganciclovir, 5 mg/kg, was administered intravenously every 24 h for 1–4 weeks after transplantation, regardless of the cytomegalovirus serostatus of recipients and donors. Subsequently, oral valganciclovir, 900 mg, was administered daily until 6 months after transplantation. For antifungal prophylaxis, voriconazole was administered, with a target trough level of 1.5–5.5 mg/dL, for 6 months postoperatively. Postoperative antibiotics were routinely adjusted on the basis of preoperative culture data obtained from the donor and the recipient bronchus.

### Assessment and management of airway complications

Airway complications were evaluated with flexible bronchoscopy, which was performed only when airway complications were suspected on the basis of clinical symptoms, deterioration in lung function, and abnormal findings on chest radiographs or computed tomographic images. We reviewed bronchoscopy reports and pictures, and airway complications were classified into five categories: bronchial stenosis, ischemia, necrosis, dehiscence, and malacia^6^. Airway complications were also evaluated with the MDS classification to standardize the description of bronchoscopic findings^24^.

Treatment plans, such as conservative treatment and bronchoscopic intervention, were also determined at the time of diagnosis. The attending physician and the interventional radiologist or pulmonologist discussed the indication for balloon dilatation or bronchial stent insertion and decided on the treatment plan. Bronchoscopic intervention, including balloon dilatation and stent insertion, was performed with flexible or rigid bronchoscopes.

### Statistical analysis

Continuous variables were calculated as means ± standard deviations, and categorical variables were calculated as percentages. We performed Student’s *t* test or the Mann–Whitney *U* test to compare continuous data and the chi-square test or Fisher’s exact test to compare categorical data. We used Cox proportional hazards regression to find risk factors for airway complications. Variables with *P* values of < 0.2 in univariable analysis were included in the multivariable analysis. All two-tailed *P* values of < 0.05 were considered statistically significant. We used the Kaplan–Meier method to plot survival curves and the Gehan–Breslow–Wilcoxon test to compare them. To perform the statistical analyses, we used IBM SPSS Statistics, version 20.0 (IBM Corporation, Armonk, NY, USA).

## Results

### Characteristics of the study population

We reviewed the records of 137 patients, of whom 89 (65.0%) were men and 48 (35.0%) were women; the median follow-up duration was 20 months (interquartile range, 7–42 months). The mean age of the patients was 53.0 ± 12.1 years.

The diseases that necessitated transplantation included idiopathic pulmonary fibrosis (IPF) (n = 60, 43.8%), non-IPF interstitial lung disease (n = 44, 32.1%), bronchiolitis obliterans (n = 7, 5.1%), and pulmonary hypertension (n = 6, 4.4%). Postoperative airway complications occurred in 30 patients (21.9%).

### Comparison of characteristics of patients with and without airway complications

The baseline characteristics of patients with and without airway complications are listed in Table 1. Most baseline characteristics of the two groups did not differ significantly; however, the proportion with a BMI of ≥ 25 kg/m^2^ was significantly higher among the patients with airway complications (40%) than among those without airway complications (20.6%; *p* = 0.029). Other characteristics, including donor-related variables, preoperative infectious episodes, and size mismatching, did not differ significantly between the two groups.

**Table 1:** Comparison of baseline characteristics of transplant recipients with and without airway complications.

Characteristic	Total	Airway complications	No airway complications	*P* value
Number of recipients	137	30	107	
Mean recipient age, years	53.0 ± 12.1	54.9 ± 10.0	52.5 ± 12.6	0.275
Male sex	89 (65.0)	22 (73.3)	67 (62.6)	0.277
BMI ≥ 25 kg/m^2^	34 (24.8)	12 (40.0)	22 (20.6)	0.029
Mean donor age, years	39.8 ± 11.2	38.0 ± 12.3	40.3 ± 10.8	0.320
Number of male donors	86 (62.8)	20 (66.7)	66 (61.7)	0.618
Diagnosis				0.616
IPF	60 (43.8)	15 (50.0)	45 (42.1)	
Non-IPF ILD	44 (32.1)	9 (30.0)	35 (32.7)	
Pulmonary hypertension	6 (4.4)	2 (6.7)	4 (3.7)	
Bronchiolitis obliterans	7 (5.1)	0 (0.0)	7 (6.5)	
Others	20 (14.6)	4 (13.3)	16 (15.0)	
Diabetes	30 (21.9)	7 (23.3)	23 (21.5)	0.830
Ever smoked	62 (45.3)	17 (56.7)	45 (42.1)	0.155
Preexisting pulmonary hypertension	68 (49.6)	15 (50.0)	53 (49.5)	0.964
Preoperative infection	41 (29.9)	11 (36.7)	30 (28.0)	0.362
*Pseudomonas aeruginosa* colonization	7 (5.1)	3 (10.0)	4 (3.7)	0.177
*Aspergillus* colonization	14 (10.2)	4 (13.3)	10 (9.3)	0.507
Size mismatching	49 (35.8)	13 (43.3)	36 (33.6)	0.328
Prior thoracic surgery	55 (40.1)	12 (40.0)	43 (40.2)	0.985
Receipt of a donor lobectomy specimen before transplantation	4 (2.9)	2 (6.7)	2 (1.9)	0.209
First lung transplanted, Rt	64 (46.7)	15 (50.0)	49 (45.8)	0.683

Data are presented as means ± standard deviations, or number (%).

*BMI* Body mass index, *IPF* Idiopathic pulmonary fibrosis, *non-IPF ILD* Non–idiopathic pulmonary fibrosis interstitial lung disease.

Perioperative management of patients with and without airway complications is described in Table 2. No perioperative variables differed significantly between the two groups, but the mean duration of postoperative mechanical ventilation (52.1 vs. 17.3 days; *p* = 0.089) and length of postoperative ICU stay (60.2 vs. 24.5 days; *p* = 0.079) were longer for patients with airway complications than for those without airway complications, with marginal significance. The proportions of patients who received preoperative mechanical ventilation and mean total ischemic times did not differ significantly between the two groups.

**Table 2:** Comparison of perioperative management in transplant recipients with and without airway complications.

Characteristic	Total	Airway complications	No airway complications	*P* value
Number of recipients	137	30	107	
Preoperative steroid use	105 (76.6)	24 (80.0)	81 (75.7)	0.623
0–0.5 mg/kg	77 (56.2)	17 (56.7)	60 (56.1)	0.954
0.5–1.0 mg/kg	20 (14.6)	4 (13.3)	16 (15.0)	> 0.999
> 1.0 mg/kg	8 (5.8)	3 (10.0)	5 (4.7)	0.372
Preoperative immunosuppressant use	19 (13.9)	6 (20.0)	13 (12.1)	0.368
Preoperative mechanical ventilation	94 (68.6)	22 (73.3)	72 (67.3)	0.528
Duration of preoperative MV (days)	20.4 ± 20.2	24.8 ± 28.2	19.0 ± 17.0	0.241
Preoperative ECMO	80 (58.4)	19 (63.3)	61 (57.0)	0.535
Duration of preoperative ECMO (days)	15.2 ± 12.7	17.7 ± 14.8	14.4 ± 12.0	0.322
Ischemic time (min)	232.5 ± 114.6	248.7 ± 145.8	228.1 ± 105.0	0.395
Postoperative ECMO	11 (8.0)	4 (13.3)	7 (6.5)	0.256
Duration of postoperative ECMO (days)	7.9 ± 9.0	11.5 ± 14.4	5.9 ± 4.3	0.495
Duration of postoperative MV (days)	24.9 ± 99.1	52.1 ± 199.7	17.3 ± 37.8	0.089
Length of postoperative ICU stay (days)	32.3 ± 98.4	60.2 ± 198.2	24.5 ± 37.2	0.079

Data are presented as means ± standard deviations or numbers (%).

*ECMO* Extracorporeal membrane oxygenation, *ICU* Intensive care unit, *MV* Mechanical ventilation.

### Risk factors for airway complication

Univariable analysis demonstrated that in transplant recipients, a BMI of ≥ 25 kg/m^2^ (hazard ratio [HR], 2.485; *p* = 0.015), duration of postoperative mechanical ventilation time (HR, 1.002; *p* = 0.023), and ICU stay (HR, 1.002; *p* = 0.021) were significantly associated with airway complications. Similarly, postoperative ECMO (HR, 2.831; *p* = 0.054) and receipt of a donor lobectomy specimen before transplantation (HR, 3,122; *p* = 0.122) were marginally associated with airway complications (Table 3).

**Table 3:** Risk factors for airway complications according to Cox proportional hazards model.

Parameter	Hazard ratio	95% confidence interval	*P* value
**Univariable analysis**			
Recipient age, years	1.015	0.983–1.048	0.371
Male sex	1.420	0.632–3.190	0.396
BMI ≥ 25 kg/m^2^	2.485	1.195–5.170	0.015
Donor age, years	0.986	0.955–1.018	0.394
Male donors	1.269	0.594–2.711	0.539
Diabetes	1.024	0.439–2.388	0.956
Ever smoked	1.557	0.756–3.205	0.230
Preexisting pulmonary hypertension	0.997	0.487–2.039	0.993
Preoperative infection	1.327	0.631–2.789	0.456
*Pseudomonas aeruginosa* colonization	1.994	0.604–6.577	0.257
*Aspergillus* colonization	1.571	0.548–4.503	0.401
Preoperative steroid use	1.417	0.579–3.468	0.446
0–0.5 mg/kg	1.140	0.553–2.347	0.723
0.5–1.0 mg/kg	0.876	0.306–2.511	0.806
Preoperative steroid use	1.417	0.579–3.468	0.446
Preoperative immunosuppressant use	1.668	0.681–4.082	0.263
Preoperative MV	1.364	0.607–3.067	0.452
Duration of preoperative MV (days)	1.008	0.993–1.024	0.288
Preoperative ECMO	1.389	0.661–2.921	0.386
Duration of preoperative ECMO (days)	1.021	0.988–1.054	0.218
Ischemic time (min)	1.002	0.998–1.005	0.311
Postoperative ECMO	2.831	0.984–8.147	0.054
Duration of postoperative MV (days)	1.002	1.000–1.004	0.023
Length of postoperative ICU stay (days)	1.002	1.000–1.004	0.021
Size mismatching	1.422	0.691–2.929	0.339
Prior thoracic surgery	0.995	0.479–2.066	0.989
Receipt of a donor lobectomy specimen before transplantation	3.122	0.738–13.208	0.122
First lung transplanted, Rt	1.154	0.564–2.361	0.696
**Multivariable analysis**			
BMI ≥ 25 kg/m^2^	2.663	1.229–5.772	0.013
Postoperative ECMO	3.340	1.093–10.211	0.034
Duration of postoperative MV (days)	0.987	0.939–1.037	0.604
Length of postoperative ICU stay (days)	1.015	0.965–1.067	0.558
Receipt of a donor lobectomy specimen before transplantation	3.216	0.744–13.907	0.118

*BMI* Body mass index, *ECMO* Extracorporeal membrane oxygenation, *ICU* Intensive care unit, *MV* Mechanical ventilation.

Multivariable analysis revealed that in transplant recipients, a BMI of ≥ 25 kg/m^2^ (HR, 2.663; *p* = 0.013) remained a significant independent risk factor for airway complications, as did postoperative ECMO (HR, 3.340; *p* = 0.034). The baseline characteristics between recipients with BMIs < 25 kg/m^2^ and ≥ 25 kg/m^2^ are listed in Supplementary Table S1. Transplant recipients with BMIs of ≥ 25 kg/m^2^, in comparison with those with lower BMIs, included more patients with diabetes mellitus (32.4 vs. 18.4%; *p* = 0.029) and who used preoperative immunosuppressants (26.5 vs. 9.7%, *p* = 0.015). The patients with higher BMIs also had a shorter duration of preoperative mechanical ventilation (15.3 vs. 22.1 days; *p* = 0.049). The baseline characteristics of recipients who did and did not receive postoperative ECMO are listed in Supplementary Table S2. The proportion of men was significantly lower (36.4 vs. 67.5%; *p* = 0.039) and ischemic time was longer (332.7 vs. 224.6 min; *p* = 0.006; Supplementary Table S2) among those who received postoperative ECMO than those who did not.

### Characteristics and management of airway complications

**Figure 1:**
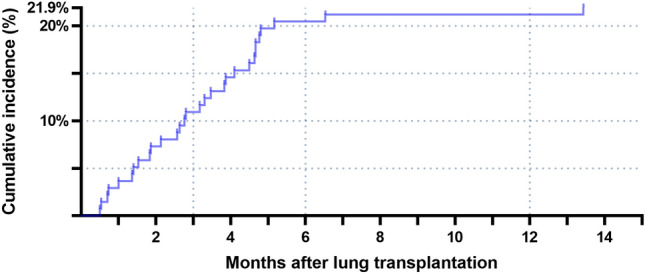
Cumulative incidence of airway complications after lung transplantation.

**Figure 2:**
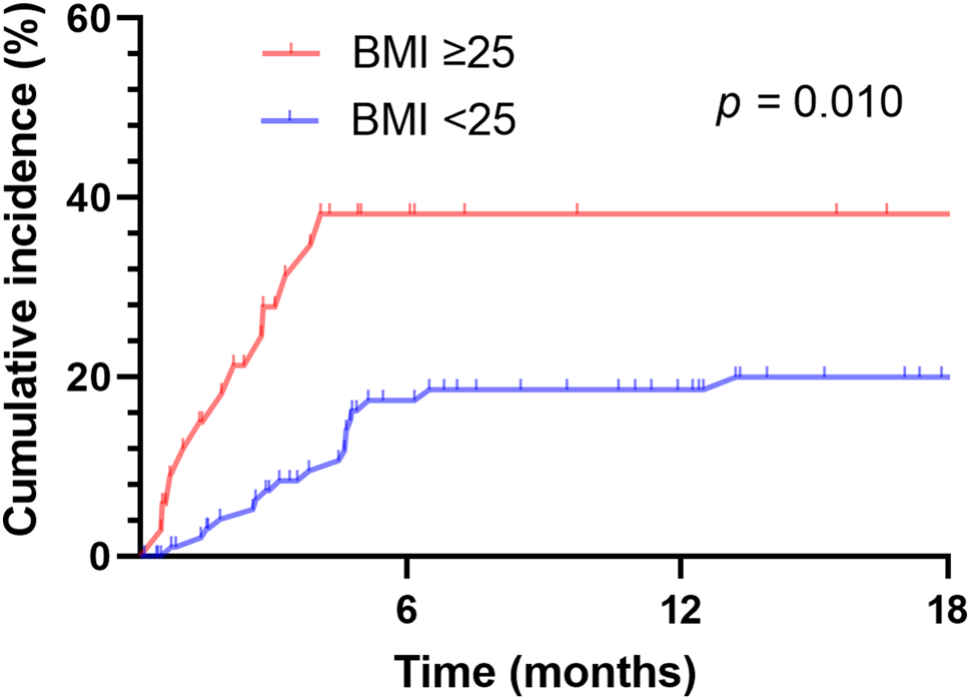
Cumulative incidence of airway complications after lung transplantation in different BMI categories.

For the 30 patients who had airway complications, the mean time between their surgeries and when complications were first detected was 3.3 ± 2.5 months; in 15 patients (50.0%), airway complications developed within the first 3 months after transplantation (Fig. 1). The cumulative incidence of airway complications based on different BMI categories was significantly higher in transplant recipients with BMIs of ≥ 25 kg/m^2^ (*p* = 0.010; Fig. 2).

Demographic characteristics of these patients and the types of management of airway complications are listed in Table 4. The most common airway complication was bronchial stenosis (n = 22), followed by bronchial ischemia (n = 6), bronchial necrosis (n = 2), and bronchomalacia (n = 2). Two of the 30 patients had two different types of airway complications: bronchial stenosis and ischemia in one and bronchial stenosis and bronchomalacia in the other. The right lung was transplanted first more often (Right 60% vs. Left 40%), and the airway complications tended to predominate on the right side. The incidence of airway complications in the lung that was transplanted first was 8.7%, in the lung that was transplanted second 6.5%, and in both 6.5%.

**Table 4:** Demographic and management of transplant recipients with airway complications.

Patient number	Age/Gender	Disease necessitating transplantation	Detection date (days after transplantation but before occurrence of airway complication)	Airway complication ((Location) MDS classification)	Intervention (total number of times)
1	56/Male	IPF	30	Stenosis ((L) M2cD3bS0)	Stenting (1)
2	29/Female	Humidifier disinfectant–related lung disease	84	Stenosis ((R) M0D3dS0)	Balloon dilatation (3), stenting (1)
3	53/Male	Drowning	140	Stenosis ((R) M2aD3aS0)	Balloon dilatation (1)
4	61/Male	IPF	83	Stenosis ((R) M2bD3bS0)	Balloon dilatation (1)
5	56/Male	CTD-related ILD	56	Stenosis ((R)M1aD3aS0)	Balloon dilatation (1)
6	53/Male	IPF	115	Stenosis ((R) M2aD3aS0)	Balloon dilatation (3), stenting (1)
7	66/Male	IPF	139	Stenosis ((R) M2bD3bS0)	None
8	66/Male	IPF	55	Necrosis ((L) M3aD0S0)	None
9	50/Male	IPF	46	Stenosis ((R) M2aD3aS0), Ischemia ((L) M2aD0S0)	Balloon dilatation (2), stenting (1)
10	56/Male	IPF	403	Stenosis ((R)M2aD3aS0)	None
11	62/Male	IPF	41	Stenosis ((L) M3aD2aS0)	Balloon dilatation (1)
12	60/Female	IPF	144	Stenosis ((R) M2bD2bS0)	Balloon dilatation (2)
13	53/Male	NSIP	104	Bronchomalacia ((R) M2aD1S0)	Balloon dilatation (1), stenting (1)
14	64/Male	IPF	79	Ischemia ((L) M3bD0S0)	None
15	62/Male	CPFE	155	Stenosis ((L) M1aD2aS0)	None
16	55/Male	IIP	77	Stenosis ((R) M2bD3bS0, (L) M2bD3bS0)	Balloon dilatation (2)
17	56/Male	IPF	196	Stenosis ((R) M1aD3aS0)	Balloon dilatation (1)
18	56/Male	IPF	140	Stenosis ((R) M1bD3bS0, (L) M1bD2bS0)	Balloon dilatation (2), stenting (1)
19	67/Male	IPF	143	Stenosis ((L) M1aD3aS0)	Balloon dilatation (4)
20	48/Female	IPAH	116	Stenosis ((R) M2aD2aS0)	Balloon dilatation (1)
21	59/Male	AIP	16	Ischemia ((R) M3aD0S0, (L) M3cD0S0)	None
22	54/Male	Bronchiectasis, COPD	135	Stenosis ((R) M0D3dS0, (L) M0D3dS0)	Balloon dilatation (6)
23	32/Male	NSIP	99	Stenosis ((R) M0cD2cS0)	Balloon dilatation (5), stenting (1)
24	38/Male	CTEPH	15	Necrosis ((L) M3cD0S0)	None
25	65/Female	IPF	123	Stenosis ((R) M1bD3bS0)	Balloon dilatation (2), stenting (1)
26	63/Female	IPF	95	Stenosis ((R) M2aD2aS0)	Balloon dilatation (2), stenting (1)
27	46/Male	OP	21	Ischemia ((R)M3aD0S0, (L) M3bD0S0)	None
28	60/Female	Bronchiectasis	22	Ischemia ((R) M3aD0S0, (L) M3aD0S0)	None
29	65/Female	CTD-related ILD	42	Stenosis ((R) M2aD3aS0, (L) M2aD0S0)	Balloon dilatation (3)
30	37/Female	NSIP	64	Stenosis ((L) M2cD2CS0), bronchomalacia ((R) M0D1S0)	Balloon dilatation (1)

*AIP* Acute interstitial pneumonia, BL Bilateral, *CPFE* Combined pulmonary fibrosis and emphysema, *CTD* Connective tissue disease, *CTEPH* Chronic thromboembolic pulmonary hypertension, *IIP* Idiopathic interstitial pneumonia, *ILD* Interstitial lung disease, *IPAH* Idiopathic pulmonary arterial hypertension, *IPF* Idiopathic pulmonary fibrosis, *L* Left, *NSIP* Nonspecific interstitial pneumonia, *OP* Organizing pneumonia, *R* Right.

Of the 30 patients with airway complications, 21 (70.0%) received bronchoscopic intervention and 9 (30.0%) did not. Balloon dilatation alone was performed in 12 patients, an average of three times. One patient underwent only stent insertion, and eight patients needed stent insertion after balloon dilatation (Table 4). No serious adverse event was associated with bronchoscopic intervention.

### Effect of airway complications on survival

**Figure 3:**
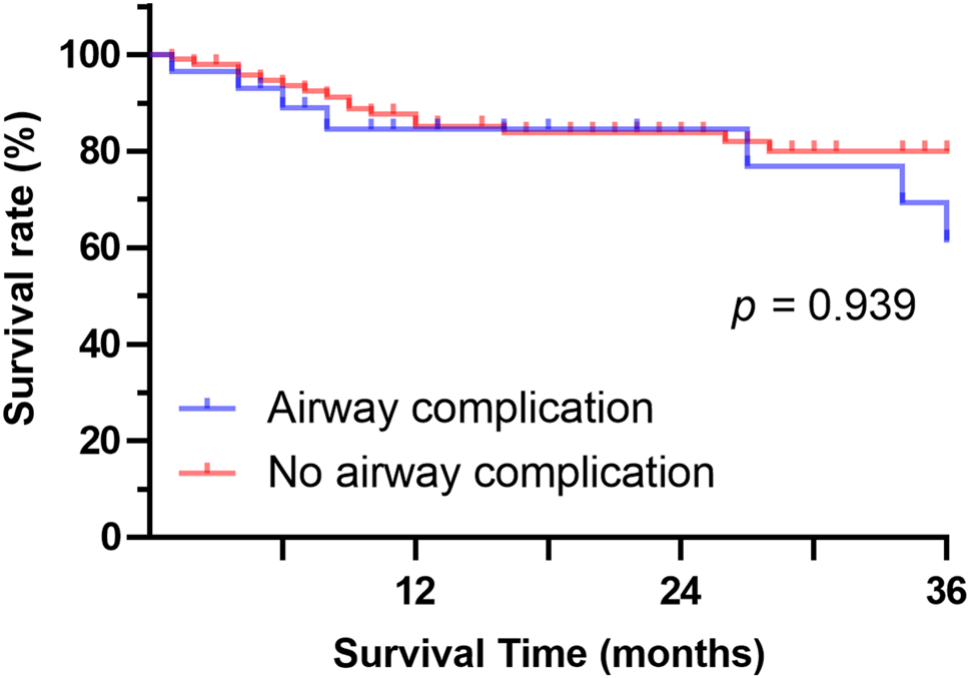
Results of Kaplan–Meier survival analysis for patients with airway complications versus those without airway complications.

The overall 1-, 2-, and 3-year survival rates after lung transplantation were 81.8, 78.9, and 72.8%, respectively. Thirty-three patients (24.1%) died during the study period. Length of survival did not differ significantly between patients with and without airway complications (mean survival time, 6.5 years vs. 7.6 years, respectively; *p* = 0.939; Fig. 3).

## Discussion

We found that airway complications after lung transplantation occurred in one fifth of our patients. Obesity of recipients and postoperative treatment with ECMO seemed to be associated with airway complications. Approximately 70.0% of patients with airway complications received bronchoscopic interventions. Length of survival did not differ significantly between patients who had airway complications and those who did not.

Airway complications are associated with significant rates of morbidity in patients who receive lung transplants^8,15^. According to previous studies, the incidence of airway complications varied between 2 and 30%^12,19,25,26^, which was comparable with the rate in our study. Additionally, a stenosis was the most common complication in most studies^19,20,27,28^, which was also consistent with our results.

Although risk factors for airway complications in patients with lung transplantation have been studied^13,14,17,18^, the results have not been consistent. Several reports suggested that bronchial ischemia caused by impaired bronchial blood supply during surgery might be associated with airway complications^29,30^. Results of other studies have suggested that donor–recipient height mismatch, primary graft dysfunction, microbiologic infection (especially with *Aspergillus fumigatus*), use of sirolimus in the early postoperative period, and prolonged ventilator care are also associated with airway complications^13,19,31^. Of note is our finding that a higher BMI of recipients and postoperative treatment with ECMO were associated with airway complications after lung transplantation. Previous studies have shown that obesity of recipients was associated with primary graft dysfunction^32,33^, and several mechanisms have been suggested, such as the technical difficulty of surgery, longer ischemic time, and decreased respiratory compliance. These disadvantages might prompt the development of airway complications. Another possible link between obesity and airway complications is the release of proinflammatory cytokines from adipose tissue in obesity, which may negatively affect bronchial healing^34^. Additionally, ECMO is associated with inflammatory reactions, such as activation of the complement and contact systems, which may lead to disruption of microcirculation and endothelial injury^35^. These reactions might impair bronchial healing and result in airway complications. Another possible mechanism linking postoperative treatment with ECMO and the development of airway complications is that one of the reasons for postoperative treatment of ECMO is primary graft dysfunction, which has been considered as a risk factor for airway complications^12^. Several complications of postoperative ECMO support may also affect the occurrence of airway complications^36^.

In our study, approximately 70.0% of patients with airway complications received bronchoscopic intervention, such as balloon dilatation and stent insertion. Although no “gold standard” has been established for the management of airway complications, several methods of treating them have been proposed, especially for airway stenosis^6,37–39^. As in previous reports^6,37,39^, out of 30 patients, 12 (40.0%) received repeated balloon dilatations and 8 (26.7%) required stent insertion for refractory airway stenosis. No serious adverse events were directly related to these procedures; therefore, bronchoscopic intervention is safe and can be useful for treating airway stenosis. However, in the case of complications other than airway stenosis, it was difficult to identify optimal treatment because the number of complications was relatively small, as in previous reports. Further research is needed to establish effective therapeutic strategies for these complications.

Previous studies have yielded conflicting data about the effect of airway complications on survival. Awori et al.^8^ demonstrated that survival rates were significantly lower among patients with airway complications than among those without. Similarly, an analysis of 312 patients after transplantation revealed a significant increase in mortality among patients with airway complications^37^. On the other hand, Yserbyt et al.^18^ reported no statistically significant difference in survival between patients who did and did not have airway complications. A retrospective review of 983 lung transplant recipients demonstrated no association between airway complications and survival^40^. In our study, we also observed no statistical differences in survival rates between patients with and those without airway complications. A possible mechanism of these results might be the appropriate management of airway stenosis, which was the most common airway complication.

This study had several limitations. First, it was a retrospective single-center study, and the results cannot be generalized. Techniques of surgeons may vary, and the variations may be immeasurable over time and with experience. Second, the study population did not reflect some characteristics of lung transplant recipients worldwide, including indications and status at the time of transplantation, due to local eligibility rules^41^. For instance, COPD, which has been the second most frequent indication for lung transplantation worldwide, accounted for only small percentage of our study population. In addition, most of the study population experienced pre-transplant mechanical ventilation, suggesting that urgent lung transplantations were performed in most cases. Owing to characteristics of the patients, it might be difficult to generalize our findings. Third, as with the diagnostic definition, the treatment of airway complications after lung transplantation has not yet been standardized; therefore, the role of intervention is not clear. Fourth, early and asymptomatic complications may have been missed because regular bronchoscopy was not performed after transplantation. The actual incidence of airway complications may have been higher than the rate we calculated. Finally, other crucial clinical parameters such as occurrence of infection, serial changes of pulmonary function test, and quality of life were not considered in the analysis according to the airway complication. Despite these limitations, our study revealed not only the incidence of airway complications after lung transplantation but also their characteristics, clinical course, and subsequent management.

In conclusion, our study showed that airway complications occurred in one fifth of lung transplant recipients. Because obesity of recipients and postoperative ECMO support are associated with airway complications, close monitoring is necessary in such cases. Although optimal management of these patients has not been elucidated, bronchoscopic intervention might be useful, especially for bronchial stenosis.

## Supplementary Information

Supplementary Information.
