# Supplementary material for: Incidence, risk factors, and clinical characteristics of airway complications after lung transplantation
Source: Sci Rep. 2023 Jan 12;13:667. doi: 10.1038/s41598-023-27864-1 (PMC9837050; doi:10.1038/s41598-023-27864-1)
Supplement: Supplementary file 1 — Supplementary Information. [file 41598_2023_27864_MOESM1_ESM.docx]

**Supplementary Information**

**e-Table 1.** Comparison of characteristics between patients with BMI ≥25 and patients with BMI <25

| Characteristic | Total | BMI ≥25 | BMI <25 | *P* value |
| --- | --- | --- | --- | --- |
| Patient numbers | 137 | 34 (24.8) | 103 (75.2) |  |
| Mean recipient age, years | 53.0 ± 12.1 | 50.9 ± 14.0 | 53.8 ± 11.4 | 0.286 |
| Male sex | 89 (65.0) | 25 (73.5) | 64 (62.1) | 0.227 |
| Mean donor age, years | 39.8 ± 11.2 | 38.6 ± 12.4 | 40.3 ± 10.8 | 0.446 |
| Number of male donors | 86 (62.8) | 20 (58.8) | 66 (64.1) | 0.583 |
| Diagnosis |  | | | 0.783 |
| IPF | 60 (43.8) | 14 (41.2) | 46 (44.7) |  |
| Non-IPF ILD | 44 (32.1) | 12 (35.3) | 32 (31.1) |  |
| Pulmonary hypertension | 6 (4.4) | 3 (8.8) | 3 (2.9) |  |
| Bronchiolitis obliterans | 7 (5.1) | 1 (2.9) | 6 (5.8) |  |
| Others | 20 (14.6) | 4 (11.8) | 16 (15.5) |  |
| Diabetes | 30 (21.9) | 11 (32.4) | 19 (18.4) | 0.029 |
| Ever smoked | 62 (45.3) | 15 (44.1) | 47 (45.6) | 0.878 |
| Preexisting pulmonary hypertension | 68 (49.6) | 15 (44.1) | 53 (51.5) | 0.458 |
| Preoperative infection | 41 (29.9) | 9 (26.5) | 32 (31.1) | 0.612 |
| *Pseudomonas aeruginosa* colonization | 7 (5.1) | 0 (0.0) | 7 (6.8) | 0.193 |
| *Aspergillus* colonization | 14 (10.2) | 3 (8.8) | 11 (10.7) | >0.999 |
| Preoperative steroid use | 105 (76.6) | 28 (82.4) | 77 (74.8) | 0.364 |
| 0–0.5 mg/kg | 77 (56.2) | 19 (55.9) | 58 (56.3) | 0.965 |
| 0.5–1.0 mg/kg | 20 (14.6) | 8 (23.5) | 12 (11.7) | 0.099 |
| >1.0 mg/kg | 8 (5.8) | 1 (2.9) | 7 (6.8) | 0.679 |
| Preoperative immunosuppressant use | 19 (13.9) | 9 (26.5) | 10 (9.7) | 0.015 |
| Preoperative MV | 94 (68.6) | 24 (70.6) | 70 (68.0) | 0.775 |
| Duration of preoperative MV (days) | 20.4 ± 20.2 | 15.3 ± 10.5 | 22.1 ± 22.4 | 0.049 |
| Preoperative ECMO | 80 (58.4) | 23 (67.6) | 57 (55.3) | 0.207 |
| Duration of preoperative ECMO (days) | 15.2 ± 12.7 | 12.1 ± 8.7 | 16.5 ± 13.9 | 0.096 |
| Ischemic time (min) | 232.5 ± 114.6 | 249.5 ± 145.6 | 227.1 ± 103.0 | 0.331 |
| Postoperative ECMO | 11 (8.0) | 2 (5.9) | 9 (8.7) | 0.597 |
| Duration of postoperative ECMO (days) | 7.9 ± 9.0 | 19.0 ± 19.8 | 5.4 ± 3.9 | 0.509 |
| Duration of postoperative MV | 24.9 ± 99.1 | 49.2 ± 187.6 | 16.9 ± 38.1 | 0.327 |
| Length of postoperative ICU stay | 32.3 ± 98.4 | 56.2 ± 186.3 | 24.5 ± 37.7 | 0.331 |
| Prior thoracic surgery | 55 (40.1) | 14 (41.2) | 41 (39.8) | 0.888 |
| Size mismatching | 49 (35.8) | 14 (41.2) | 35 (34.0) | 0.448 |
| Receipt of a donor lobectomy specimen before transplantation | 4 (2.9) | 1 (2.9) | 3 (2.9) | > 0.999 |
| First lung transplanted, Rt. | 64 (46.7) | 16 (47.1) | 48 (46.6) | 0.963 |

Data are presented as mean ± standard deviation, median [interquartile range], or number (%)

BMI; body mass index, ECMO; extracorporeal membrane oxygenation, ICU; intensive care unit, MV; mechanical ventilation

**e-Table 2.** Comparison of characteristics between patients with postoperative ECMO and patients without postoperative ECMO

| Characteristic | Total | Postoperative ECMO | No postoperative ECMO | *P* value |
| --- | --- | --- | --- | --- |
| Patient numbers | 137 | 11 | 126 |  |
| Mean recipient age, years | 53.0 ± 12.1 | 50.8 ± 11.7 | 53.2 ± 12.2 | 0.528 |
| Male sex | 89 (65.0) | 4 (36.4) | 85 (67.5) | 0.039 |
| BMI ≥25 kg/m^2^ | 34 (24.8) | 2 (18.2) | 32 (25.4) | 0.597 |
| Mean donor age, years | 39.8 ± 11.2 | 37.3 ± 13.2 | 40.1 ± 11.0 | 0.431 |
| Number of male donors | 86 (62.8) | 9 (81.8) | 77 (61.1) | 0.175 |
| Diagnosis |  | | | 0.930 |
| IPF | 60 (43.8) | 3 (27.3) | 57 (45.2) |  |
| Non-IPF ILD | 44 (32.1) | 4 (36.4) | 40 (31.7) |  |
| Pulmonary hypertension | 6 (4.4) | 3 (27.3) | 3 (2.4) |  |
| Bronchiolitis obliterans | 7 (5.1) | 1 (9.1) | 6 (4.8) |  |
| Others | 20 (14.6) | 0 (0.0) | 20 (15.9) |  |
| Diabetes | 30 (21.9) | 1 (9.1) | 29 (23.0) | 0.286 |
| Ever smoked | 62 (45.3) | 3 (27.3) | 59 (46.8) | 0.213 |
| Preexisting pulmonary hypertension | 68 (49.6) | 7 (63.6) | 61 (48.4) | 0.333 |
| Preoperative infection | 41 (29.9) | 2 (18.2) | 39 (31.0) | 0.377 |
| *Pseudomonas aeruginosa* colonization | 7 (5.1) | 0 (0.0) | 7 (5.6) | >0.999 |
| *Aspergillus* colonization | 14 (10.2) | 3 (27.3) | 11 (8.7) | 0.086 |
| Preoperative steroid use | 105 (76.6) | 9 (81.8) | 96 (76.2) | >0.999 |
| 0–0.5 mg/kg | 77 (56.2) | 6 (54.5) | 71 (56.3) | >0.999 |
| 0.5–1.0 mg/kg | 20 (14.6) | 1 (9.1) | 19 (15.1) | >0.999 |
| >1.0 mg/kg | 8 (5.8) | 2 (18.2) | 6 (4.8) | 0.126 |
| Preoperative immunosuppressant use | 19 (13.9) | 2 (18.2) | 17 (13.5) | 0.667 |
| Preoperative MV | 94 (68.6) | 9 (81.8) | 85 (67.5) | 0.327 |
| Duration of preoperative MV (days) | 20.4 ± 20.2 | 16.6 ± 12.6 | 20.8 ± 20.8 | 0.552 |
| Preoperative ECMO | 80 (58.4) | 8 (72.7) | 72 (57.1) | 0.316 |
| Duration of preoperative ECMO (days) | 15.2 ± 12.7 | 13.5 ± 11.5 | 15.4 ± 12.9 | 0.693 |
| Ischemic time (min) | 232.5 ± 114.6 | 322.7 ± 166.7 | 224.6 ± 106.2 | 0.006 |
| Duration of postoperative MV | 24.9 ± 99.1 | 45.6 ± 96.4 | 23.1 ± 99.5 | 0.472 |
| Length of postoperative ICU stay | 32.3 ± 98.4 | 53.5 ± 94.7 | 30.5 ± 98.8 | 0.459 |
| Prior thoracic surgery | 55 (40.1) | 5 (45.5) | 50 (39.7) | 0.755 |
| Size mismatching | 49 (35.8) | 2 (18.2) | 47 (37.3) | 0.327 |
| Receipt of a donor lobectomy specimen before transplantation | 4 (2.9) | 0 (0.0) | 4 (3.2) | 0.550 |
| First lung transplanted, Rt. | 64 (46.7) | 3 (27.3) | 61 (48.4) | 0.178 |

Data are presented as mean ± standard deviation, median [interquartile range], or number (%)

BMI; body mass index, ECMO; extracorporeal membrane oxygenation, ICU; intensive care unit, MV; mechanical ventilation
